# Comparison of breast cancer prognostic tests CanAssist Breast and Oncotype DX

**DOI:** 10.1002/cam4.3495

**Published:** 2020-10-07

**Authors:** Aditya K. Sengupta, Aparna Gunda, Sukriti Malpani, Chandra Prakash V. Serkad, Chetana Basavaraj, Ashok Bapat, Manjiri M. Bakre

**Affiliations:** ^1^ OncoStem Diagnostics Bangalore Karnataka India; ^2^ Virtua Hospital Voorhees NJ USA

## Abstract

**Background:**

CanAssist Breast (CAB) is a prognostic test for early stage hormone receptor‐positive (HR+), human epidermal growth factor receptor 2 negative (HER2−) breast cancer patients, validated on Indian and Caucasian patients. The 21‐gene signature Oncotype DX (ODX) is the most widely used commercially available breast cancer prognostic test. In the current study, risk stratification of CAB is compared with that done with ODX along with the respective outcomes of these patients.

**Methods:**

A cohort of 109 early stage breast cancer patients who had previously taken the ODX test were retested with CAB, and the results respectively compared with old cut‐offs of ODX as well as cut‐offs suggested by TAILORx, a prospective randomized trial of ODX. Distant metastasis‐free survival after 5 years was taken as the end point.

**Results:**

CanAssist Breast stratified 83.5% of the cohort into low‐risk and 16.5% into high‐risk. With the TAILORx cut‐offs, ODX stratified the cohort into 89.9% low‐risk and 10.1% into high‐risk. The low, intermediate, and high‐risk groups with ODX old cut‐offs were 62.4%, 31.2%, and 6.4%, respectively. The overall concordance of CAB with ODX using both cut‐offs is 75%‐76%, with ~82%‐83% concordance in the low‐risk category of these tests. The NPV of the low‐risk category of CAB was 93.4%, and of ODX with TAILORx cut‐offs was 91.8% and 89.7% with old cut‐offs.

**Conclusions:**

Compared to the concordance reported for other tests, CAB shows high concordance with ODX, and in addition shows comparable performance in the patient outcomes in this cohort. CAB is thus an excellent and cost‐effective alternative to ODX.

## Graphical abstract

Risk stratifications and outcomes of hormone receptor‐positive/human epidermal growth factor receptor 2 negative breast cancer prognostic tests CanAssist Breast (CAB) and Oncotype DX (ODX) were compared in a cohort of 109 patients who had previously taken the ODX test. Using old cut‐offs of ODX as well as those suggested by the TAILORx study, the overall concordance of CAB with ODX was 75%‐76%, with ~82%‐83% concordance in the low‐risk category. The NPV of the low‐risk category of CAB was 93.4% and of ODX was 89.7%‐91.8%. CAB is thus an excellent and cost‐effective alternative to ODX.

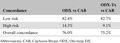

## 1 BACKGROUND

Hormone receptor‐positive (HR+) breast cancer patients are treated with adjuvant endocrine therapy after surgery and if detected at an early stage, and can avoid chemotherapy.^1^, ^2^ Around 15% of these early stage HR+/human epidermal growth factor receptor 2 negative (HER2−) patients are typically at a high‐risk for distant recurrence, and require adjuvant chemotherapy.^1^ Thus, assessment of the risk of distant recurrence in these patients is crucial for determining the mode of treatment.

Various prognostic tools have been used to assess which patients could be spared or would benefit from adjuvant chemotherapy, to respectively avoid over‐ and under‐treatment. These include clinical features, histological features, and multigene tests. Some of the prognostic tools do not go beyond the standard biomarkers such as estrogen receptor, progesterone receptor, HER2, and Ki‐67. Several gene‐expression prognostic assays focus primarily on proliferation. However, the biology of disease progression and distant metastasis is complex and involves the tumor microenvironment and cross talk between various signaling pathways, including cancer stem cell self‐renewal, loss of cell adhesion, epithelial‐mesenchymal transition, mesenchymal‐epithelial transition, drug transporters, etc.^3^, ^4^, ^5^, ^6^, ^7^, ^8^, ^9^, ^10^ In addition, transcriptional abundance of a gene does not necessarily correlate with its post‐translational modifications of proteins are not captured by gene‐expression analysis.^11^ Thus, many of the genomics‐based prognostic tools may not capture the true aggressiveness of the tumor.

A number of available prognostic tests predict risk of recurrence for early stage HR+ breast cancer based on multigene expression (Oncotype DX [ODX], MammaPrint, Breast Cancer Index), or a combination of multigene expression and clinical parameters (Prosigna, EndoPredict‐EPclin, CanAssist Breast [CAB]).^12^, ^13^, ^14^, ^15^, ^16^, ^17^, ^18^, ^19^

Oncotype DX is perhaps the most widely used of the commercially available breast cancer prognostic test. It takes into account a 21 gene signature to calculate a recurrence score (RS), and thus, stratifies patients into low‐risk (RS < 18), intermediate‐risk (RS 18‐30), and high‐risk (RS > 30) of recurrence.^12^, ^20^, ^21^, ^22^, ^23^, ^24^ Interestingly, it was recently shown by the prospective TAILORx study of ODX that while some younger women (≤50 years) with RS 16‐25 did benefit from chemotherapy, women above 50 years with RS ≤ 25 did not benefit from chemotherapy.^25^

CanAssist Breast combines the immunohistochemistry (IHC) data of five biomarkers with three clinical parameters (tumor size, grade, and node status) with a machine learning‐based statistical algorithm to calculate a risk score. CAB thus stratifies early stage HR+ HER2− breast cancer patients into low‐risk or high‐risk for distant recurrence.^17^, ^18^, ^19^ CAB has been retrospectively validated in a mixed cohort of Asian and Caucasian patients and is being used by clinicians in India.^19^, ^26^

A few studies have compared the available prognostic tests.^27^, ^28^, ^29^, ^30^, ^31^, ^32^, ^33^, ^34^, ^44^ ODX, MammaPrint, Prosigna, MammaTyper, IHC4‐AQUA, and IHC4 have been compared in the OPTIMA prelim trial which found that only 39.4% tumors were classified as either low/intermediate‐risk or high‐risk by all the tests.^33^

In this study, we compare the risk stratification and outcomes of a cohort of 109 early stage HR+ Her2/neu‐negative breast cancer patients by CAB and ODX. We find the overall concordance of stratification by CAB with that by ODX to be 75%‐76% for two different cut‐offs used by ODX, while the concordance in the low‐risk categories of these tests is ~82%‐83%. The outcomes of the tests were also similar as measured by the NPV of the low‐risk categories: 93.4% for CAB and 89.7%‐91.8% for ODX.

## 2 METHODS

### 2.1 Ethics approval

This study was approved by the institutional review board and/or Ethics and Scientific Committees of participating hospitals. All studies were performed with the approval of the Bangalore Ethics Committee (ECR/87/Indt/KA/2013) and in accordance with the Declaration of Helsinki. The study was performed as per the committee recommendation. Patient information was anonymized prior to analysis.

### 2.2 Patient samples

As a part of validation studies for CAB, ~1300 patient samples along with outcome data from early stage (I‐IIIA) HR+/HER2− invasive breast carcinoma patients, with a minimum follow‐up of 5 years from diagnosis, were collected retrospectively from various hospitals and biorepositories from India, United States, and Europe between 2011 and 2019 (19, 26, and unpublished data). Among these patient samples, those for which ODX test had already been conducted as a part of their treatment planning, were used in the current comparison study between CAB and ODX. The patient cohort in this study consisted of 109 samples from the above validation set, obtained between 2012 and 2019 from Virtua Hospital Voorhees, USA (*n* = 92), Sapien Biosciences, India (*n* = 9), Rajiv Gandhi Cancer Institute & Research Center, India (*n* = 4), Valle de Hebrón Instituto de Oncología, Spain (*n* = 3), and Manipal Hospital, India (*n* = 1). Seventy‐eight of these samples had been a part of previously published CAB validation studies.^19^ Given that ODX is unaffordable for most Indian breast cancer patients, very few Indian samples fit in this category, and ODX was not so commonly used in Spain during the time period of the study, thus, only 14 Indian samples along with three from Spain were included in the current study without bias to ethnicity or nationality. Informed Consent was based on country‐specific guidelines. Informed Consent was available for all patients of Virtua Hospital. For the other participating centers, the ethics committee waived the requirement for informed consent based on local guidelines considering the study was anonymized, retrospective, and non‐interventional in nature. Formalin‐fixed paraffin‐embedded (FFPE) tumor specimens from these patients along with patient and treatment follow‐up details were obtained from the respective treating hospital or biorepository. CanAssist Breast was performed on the FFPE tumor specimens from these patients.

### 2.3 ODX test scores and risk categories

The available RS data from ODX tests previously taken by these patients were used for our comparison purposes in two ways. First, the original risk categorization cut‐offs of RS < 18 for low‐risk, RS 18‐30 for intermediate‐risk, and RS > 30 for high‐risk were used. The second comparison was based on the TAILORx study cut‐offs^13^, ^23^ and the consequent test information provided in the website of Exact Sciences/Oncotype IQ,^24^ of low‐risk (RS ≤ 25) and high‐risk (RS > 25).

### 2.4 CAB testing

CanAssist Breast assay was performed on FFPE blocks as described earlier.^17^, ^19^ Briefly, IHC grading for CAB biomarkers along with tumor size, grade and node status were used to arrive at a CAB risk score between 0 and 100 using the CAB algorithm. A cut‐off of 15.5 is used to stratify the patients into low‐risk (≤15.5) and high‐risk (>15.5) categories of distant recurrence.

### 2.5 End point

Distant metastasis‐free survival was calculated as the time between the date of diagnosis of cancer and the last date of follow‐up in case of no distant recurrence with a minimum period of 5 years, which was taken as the end point of the study. Contra‐lateral, locoregional, or ipsilateral recurrences were not considered as distant recurrence.

### 2.6 Data analysis

Concordance of CAB with ODX was calculated for low‐risk category by calculating the percentage of patients classified as low ‐risk by ODX that CAB also stratifies as low‐risk. Concordance was similarly calculated for the high‐risk category. Total concordance was calculated as the percentage of patients classified by CAB into the identical risk categories as ODX. Negative predictive value (NPV) for a test for 95% CI was calculated using MedCalc.

## 3 RESULTS

### 3.1 Study cohort

The study cohort consisted of 109 patients who had previously taken the ODX test as prescribed by their treating physicians for assisting them to take treatment decisions. While ODX data of the 78 samples that was a part of the previous CAB validation study^19^ had been available, they had not been compared with CAB or outcomes in the previous study.^19^ The median age was 59 years (range 26‐74 years) with 73.4% above 50 years old. Ninety‐three patients (85.3%) had node negative disease while a majority of the tumors were grade 2 (62.4%) and T1 (69.7%) (Table 1). Distant recurrence was seen in 9 (8.3%) of the patients. Radiation therapy information was available for 101 patients; of these 77 (76%) had received radiation therapy. All patients had received endocrine therapy, while 33 (30.3%) received chemotherapy.

**TABLE 1:** Description of the patient cohort. Percentages of distant recurrences are calculated with respect to the number of patients within corresponding category.

Parameter	Number (*n* = 109)	Distant recurrence
Age		
≤50	29 (26.6%)	3 (10.3%)
>50	80 (73.4%)	6 (7.5%)
Tumor size		
T1	76 (69.7%)	4 (5.3%)
T2	33 (30.3%)	5 (15.2%)
Grade		
1	22 (20.2%)	2 (9.1%)
2	68 (62.4%)	5 (7.4%)
3	19 (17.4%)	2 (10.5%)
Node status		
N0	93 (85.3%)	7 (7.5%)
N1	15 (13.8%)	2 (13.3%)
N2	1 (0.9%)	0 (0%)
Therapy		
Endocrine therapy alone	76 (69.7%)	7 (9.2%)
Endocrine therapy + Chemotherapy	33 (30.3%)	2 (6.1%)
Distant recurrence		
No recurrence	100 (91.7%)	N.A.
Recurrence	9 (8.3%)	N.A.

### 3.2 ODX test results and chemotherapy

The ODX test performed on the cohort had used the old cut‐offs to assign risk categories and plan the subsequent treatment. The originally assigned ODX results stratified 68 (62.4%) patients in the low‐risk category, 34 (31.2%) in intermediate category and 7 (6.4%) patients in the high‐risk category (Table 2). Of the 68 ODX low‐risk patients, 6 received chemotherapy and none of them recurred. However, 7 (11.3%) of the 62 ODX low‐risk category that had received endocrine therapy alone recurred within 5 years. Twenty patients from the intermediate‐risk category received chemotherapy, of whom two showed distant recurrence within 5 years. None recurred among those from the intermediate category that had received endocrine therapy alone. All 7 ODX high‐risk patients received chemotherapy, and none recurred within 5 years (Table 2). Since the original ODX recommendations were valid for node negative patients, the 93 node negative patients were separately analyzed (Table S1). Sixty‐one (65.6%) node negative patients were assigned to the ODX low‐risk category and 5 (8.2%) of these who had received endocrine therapy alone showed distant recurrence within 5 years (Table S1). Two of the 27 node negative patients assigned to intermediate‐risk category recurred, despite having received chemotherapy. All five patients assigned to the ODX high‐risk category received chemotherapy and none recurred (Table S1).

**TABLE 2:** Comparison of risk stratification and outcomes by ODX (original cut‐offs and TAILORx cut‐offs) and CAB. Percentages of patients with or without distant recurrence are expressed with respect to the total number in the respective subcategory. Patients who received endocrine therapy alone are designated as ET and those that received both endocrine therapy and chemotherapy are designated as ET + CT.

Risk category	*n* = 109	ODX (original cut‐offs)			ODX (TAILORx cut‐offs)			CAB		
		Total	Non‐recurred	Recurred	Total	Non‐recurred	Recurred	Total	Non‐recurred	Recurred
Low‐risk	Total	68	61 (89.7%)	7 (10.3%)	98	90 (91.8%)	8 (8.2%)	91	85 (93.4%)	6 (6.6%)
	ET	62	55 (88.7%)	7 (11.3%)	76	69 (90.8%)	7 (9.2%)	62	58 (93.5%)	4 (6.5%)
	ET + CT	6	6 (100%)	0 (0%)	22	21 (95.5%)	1 (4.5%)	29	27 (93.1%)	2 (6.9%)
Intermediate‐risk	Total	34	32 (94.1%)	2 (5.9%)	NA	NA	NA	NA	NA	NA
	ET	14	14 (100%)	0 (0%)	NA	NA	NA	NA	NA	NA
	ET + CT	20	18 (90%)	2 (10%)	NA	NA	NA	NA	NA	NA
High‐risk	Total	7	7 (100%)	0 (0%)	11	10 (90.9%)	1 (9.1%)	18	15 (83.3%)	3 (16.7%)
	ET	0	0	0	0	0	0	14	11 (78.6%)	3 (21.4%)
	ET + CT	7	7 (100%)	0 (0%)	11	10 (90.9%)	1 (9.1%)	4	4 (100%)	0 (0%)

Abbreviations: CAB, CanAssist Breast; ET, endocrine therapy alone; ET + CT. both endocrine therapy and chemotherapy; ODX, Oncotype DX with old cut‐offs; ODX‐Tx, Oncotype DX with TAILORx cut‐offs.

### 3.3 ODX classes with TAILORx cut‐offs

Based on the TAILORx study and^25^, ^35^ and test information provided in the website of Exact Sciences/Oncotype IQ,^36^ the ODX RS scores were re‐stratified into two risk groups of low‐risk (RS ≤ 25) and high‐risk (RS > 25). Ninety‐eight patients (89.9%) were re‐stratified as low‐risk, while 11 as high‐risk with the new cut‐offs (Table 2). All patients of the new high‐risk group had received chemotherapy and one of these patients showed distant recurrence. Of the low‐risk group, 76 had received endocrine therapy alone, and 7 (9.2%) of these showed distant recurrence within 5 years. One patient within the low‐risk group that had received chemotherapy recurred. Since the TAILORx study was conducted on node negative patients,^25^, ^35^ we examined the 93 node negative patients with TAILORx cut‐offs (Table S1). We find that 84 (90.3%) node negative patients are assigned to low‐risk, of whom 6 (7.1%) recurred; five of these recurred patients had received only endocrine therapy. Nine node negative patients were assigned to high‐risk category, of whom all received chemotherapy, and one of these patients recurred. We also analyzed the node positive patients among the cohort (Table S1) since the website of Exact Sciences/Oncotype IQ^36^ recommends ODX with TAILORx cut‐offs for breast cancer patients up to Stage IIIA. Of the 16 node positive patients, 15 are N1, while one patient is N2—the latter had received endocrine therapy alone and did not show distant recurrence (Table 1). With TAILORx cut‐offs, ODX classified 14 of these node positive patients as low‐risk. Eight of them received endocrine therapy alone, of whom two recurred (Table S1). The rest of the six node positive patients stratified as low‐risk received chemotherapy and none recurred. Both node positive patients that stratified as high‐risk received chemotherapy and none recurred (Table S1).

### 3.4 CAB risk stratification

As described above, CAB was performed on these 109 patients, resulting in stratification of 91 patients (83.5%) into low‐risk and 18 into high‐risk, respectively (Table 2). Of the 91 CAB low‐risk patients, 29 had received chemotherapy, of whom two recurred. Of the 62 CAB low‐risk patients who had received endocrine therapy alone, four recurred (6.5%). Within the CAB high‐risk category only four had received chemotherapy, and none of these recurred. Of the 14 CAB high‐risk patients who received endocrine therapy alone, three recurred (Table 3). Both the training and validation sets for CAB had included node negative and node positive patients.^17^, ^19^ We hence separately examined the CAB categorizations for the 93 node negative and 16 node positive patients (Table S1). CAB stratifies 81 of the node negative patients as low‐risk, of whom 5 (6.2%) recurred. Fifty‐seven of these low‐risk patients had received endocrine therapy alone and 3 (5.3%) of them recurred (Table S1). Eleven of the 12 patients that CAB classified as high‐risk had received endocrine therapy alone and 2 (18.2%) of them showed distant recurrence (Table S1). These recurrence rates were similar to those previously seen: 4.7% for low‐risk, 15.6% for high‐risk in the total cohort, 4.9% and 20.0%, respectively, for low‐ and high‐risk in patients that did not receive chemotherapy.^19^ The minor differences in recurrence rates can be attributed to the small sample size of the current study.

**TABLE 3:** Comparison of original ODX categories with CAB stratification along with outcomes. Percentages of CAB numbers are expressed with respect to the corresponding ODX category. Percentages of distant recurrences are expressed with respect to corresponding ODX‐CAB common category. Note that all patients categorized as ODX high‐risk received chemotherapy.

	ODX low‐risk		ODX intermediate‐risk		ODX high‐risk		Total
	Number	Recurred	Number	Recurred	Number	Recurred	
CAB low‐risk	56 (82.4%)	4 (7.1%)	29 (85.3%)	2 (6.9%)	6 (85.7%)	0 (0%)	91
CAB high‐risk	12 (17.6%)	3 (25%)	5 (14.7%)	0 (0%)	1 (14.3%)	0 (0%)	18
Total	68 (100%)	7 (10.3%)	34 (100%)	2 (5.9%)	7 (100%)	0 (0%)	109

Abbreviations: CAB, CanAssist Breast; ODX, Oncotype DX with old cut‐offs.

### 3.5 Comparison of risk categories of ODX and CAB

Using old cut‐offs for ODX, of the 68 ODX low‐risk patients, CAB classified 56 as low‐risk, showing an 82.4% concordance in the low‐risk category (Tables 3 and 5). Of the seven ODX high‐risk patients, CAB classified 6 as high‐risk. Taken together CAB shows 82.7% concordance with ODX in the low‐risk and high‐risk classes (Tables 3 and 5). Interestingly, three patients that ODX had classified as low‐risk and had received endocrine therapy alone had been classified as high‐risk by CAB, had recurred within 5 years (Table 3; Table S2). The original ODX categorization had classified 34 (31.2%) patients as intermediate, and 20 of them had received chemotherapy. CAB classified 29 (85%) of these patients as low‐risk and five as high‐risk (Table 3). Only two of these 29 recurred, despite both patients having received chemotherapy. All five ODX intermediate/CAB high‐risk patients had received chemotherapy. Within the subcohort of 62 patients who had received endocrine therapy alone, CAB shows 82.3% concordance within the low‐risk category of ODX (Table S2). Similarly, CAB shows a concordance of 85.7% within ODX low‐risk category of the node negative patients who had received endocrine therapy alone (Table S2). Since all ODX high‐risk patients had received chemotherapy, a similar analysis for chemo‐naïve high‐risk patients could not be done.

Of the 98 patients ODX low‐risk category with TAILORx cut‐offs, 81 were stratified as low‐risk by CAB, showing 82.7% concordance within the low‐risk category (Tables 4 and 5). Of the 11 in the ODX high‐risk category with TAILORx cut‐offs, one was stratified as high‐risk by CAB, showing an 11% concordance (Tables 4 and 5). The overall concordance of CAB categories in comparison to ODX categories with TAILORx cut‐offs was thus 75.2% (Tables 4 and 5). Interestingly of the eight patients that recurred within the ODX low‐risk group with TAILORx cut‐offs, three were categorized as high‐risk by CAB. All of these three ODX low‐risk/CAB high‐risk patients had received endocrine therapy alone (Table 4; Table S2). One patient classified as ODX high‐risk but CAB low‐risk recurred despite being given chemotherapy (Tables 2 and 4). Within the subcohort of 62 patients who had received endocrine therapy alone, a concordance of 81.6% between patients classified as low‐risk by CAB and ODX with TAILORx cut‐offs (Table S2). The concordance increases slightly to 83.8% among the node negative patients who had received endocrine therapy alone (Table S2).

**TABLE 4:** Comparison of ODX with TAILORx cut‐offs (ODX‐Tx) categories with CAB stratification along with outcomes. Percentages of CAB numbers are expressed with respect to the corresponding ODX‐Tx category. Percentages of distant recurrences are expressed with respect to corresponding ODX‐Tx‐TCAB common category. Note that all patients categorized as ODX‐Tx high‐risk received chemotherapy.

	ODX‐Tx low‐risk		ODX‐Tx high‐risk		Total
	Number	Recurred	Number	Recurred	Number
CAB low‐risk	81 (82.7%)	5 (6.2%)	10 (90.9%)	1 (10.0%)	91
CAB high‐risk	17 (17.3%)	3 (17.7%)	1 (9.1%)	0 (0%)	18
Total	98 (100%)	8 (8.2%)	11 (100%)	1 (9.1%)	109

Abbreviations: CAB, CanAssist Breast; ODX‐Tx, Oncotype DX with TAILORx cut‐offs.

**TABLE 5:** Concordance of CAB with ODX with old and TAILORx cut‐offs (ODX‐Tx).

Concordance	ODX vs CAB	ODX‐Tx vs CAB
Low‐risk	82.4%	82.7%
High‐risk	14.3%	9.1%
Overall concordance	76.0%	75.2%

Abbreviations: CAB, CanAssist Breast; ODX, Oncotype DX with old cut‐offs; ODX‐Tx, Oncotype DX with TAILORx cut‐offs.

### 3.6 Performance of CAB and ODX

As noted above, CAB shows over 82.4% concordance with the low‐risk categories of ODX with old cut‐offs, and 82.7% concordance with TAILORx cut‐offs, with an overall concordance of 76% and 75.2%, respectively (Table 5). CAB thus shows good concordance with ODX, particularly in comparison to most tests for which concordance with ODX has been studied.^27^, ^28^, ^29^, ^33^, ^34^ An indication of the overall performance of all three tests can be inferred from Tables 2, 3, 4 and Tables S1 and S2. There are a total of nine patients in the cohort who had distant recurrences within 5 years of surgery. Of these, seven patients received endocrine therapy alone. While ODX categorized all of these seven into low‐risk by both cut‐offs used here, CAB classified three of the patients who had received endocrine therapy alone into high‐risk, indicating better risk prediction for these patients (Table 2; Table S2). Comparison of performance with the NPV of the low‐risk category of each of these (Table 6) shows that CAB has the highest NPV within the total cohort (93.4%), the subcohort that had received endocrine therapy alone (93.1%), as well as among the node negative patients (93.8%). Oncotype DX with TAILORx cut‐offs are the next best with 91.8%, 90.8%, and 92.8%, respectively, followed by ODX with old cut‐offs—89.9%, 88.7%, and 91.8%, respectively.

**TABLE 6:** Performance by NPV of CAB, ODX, and ODX with TAILORx cut‐offs (ODX‐Tx). Patients who received endocrine therapy alone are designated as ET, and node negative patients as N0.

NPV	Total cohort	ET	N0
CAB	93.4% (CI 89.9‐95.8)	93.6% (CI 88.3‐96.5)	93.8% (CI 90.3‐96)
ODX	89.9% (CI 85.8‐92.8)	88.7% (CI 87.5‐89.9)	91.8% (CI 87.3‐94.8)
ODX‐Tx	91.8% (CI 89.9‐93.5)	90.8% (CI 90.8‐90.8)	92.8% (CI 90.4‐94.6)

Abbreviations: CAB, CanAssist Breast; ET, endocrine therapy alone; N0, node negative; NPV, negative predictive value; ODX, Oncotype DX with old cut‐offs; ODX‐Tx, Oncotype DX with TAILORx cut‐offs.

## 4 DISCUSSION

CanAssist Breast was developed as an affordable alternative to expensive multigene prognostic tests for early stage HR+ breast cancer, particularly in developing countries in Asia.^17^, ^18^, ^19^, ^26^ The test was developed on Indian patients, and has since been validated in patients from India and United States.^17^, ^19^ Oncotype DX is one of the oldest breast cancer prognostic tests,^12^ and in many ways remains a standard bearer even in India and other developing countries where only a tiny fraction of patients can actually afford it. The current study was hence a direct retrospective comparison of CAB and ODX within a cohort of breast cancer patients who had previously taken the ODX test in connection with their treatment planning. Since all patients had detailed follow‐ups, the outcomes, that is, distant recurrences in 5 years, of these patients could be directly compared between the tests.

Since its development, following assessments by various trials and studies, notably TAILORx, the risk categorization of ODX has undergone a change, currently doing away with its previous intermediate category.^25^, ^35^, ^36^ CanAssist Breast was designed to have two unambiguous categories for risk of recurrence, low‐risk and high‐risk; chemotherapy being recommended in the latter category.^17^ In the current study, CAB risk category results have been compared with the respective low‐ and high‐risk categories of ODX with both the original and TAILORx cut‐offs, and also compared against the incidences of distant recurrence in these patients within the end point of 5 years.

While CAB was developed for both node negative and node positive patients,^17^, ^18^, ^19^ ODX was initially developed on node negative breast cancer patients and the TAILORx study also focused on node negative patients.^12^, ^25^ As noted above, the website of Exact Sciences/Oncotype IQ recommends ODX with TAILORx cut‐offs for breast cancer patients up to Stage IIIA.^36^ Numerous studies have observed the utility of ODX on node positive patients, and this aspect continues to be discussed in literature.^37^, ^38^, ^39^, ^40^, ^41^, ^42^, ^43^ Additionally, the West German Study Group Plan B trial and RxPONDER investigate the relevance of ODX scores in clinically intermediate to high‐risk early stage breast cancer patients, including in node positive patients.^38^, ^41^, ^42^, ^43^, ^45^, ^46^ We have hence included 16 node positive patients in the current study, but have also analyzed the node negative sub‐cohort separately. Also, all the node positive patients in the current cohort were prescribed ODX at least 5 years ago, and thus, we believe the inclusion of node positive patients in the current cohort is clinically relevant and of interest to practicing clinicians.

Previous comparison studies, such as by the OPTIMA prelim trial, of other breast cancer prognostic tests with ODX suggested low overall concordance (<40%) between tests, possibly because different tests employ different technologies and biomarkers.^33^ A meta‐analysis of 14 studies of at a total of 5514 patients indicated a discordance of 42%‐66% between other tests compared to ODX.^44^ From the results presented above we find that unlike most of these comparison studies of other prognostic tests with ODX, CAB performs remarkably well, with an overall concordance of 76% and 75.2%, respectively, against the original and TAILORx cut‐offs of ODX. The concordance within the low‐risk categories are higher, at 82.4% and 82.7%, respectively. The low‐risk group is the most important group in prognostic tests of this kind where the purpose is to assess if the patient can avoid chemotherapy due to low‐risk of recurrence. Indeed, a comparison of NPV for the low‐risk prediction of ODX and CAB shows that the NPV of CAB is the highest (93%) in both the total cohort as well in those patients who received endocrine therapy alone and their outcomes are thus unaffected by chemotherapy. The NPVs of ODX with TAILORx cut‐offs follow close behind with 91.8% in the total cohort and 90.7% in the patients who received endocrine therapy alone. Similarly, both cut‐offs of ODX stratified into low‐risk, the seven patients in the cohort that showed distant recurrence and had received endocrine therapy alone; however, three of these seven were classified as high‐risk by CAB. Concordance of CAB with ODX within the high‐risk category is 14.3% and 9.1%, respectively, for the original cut ODX cut‐offs and the TAILORx cut‐offs. On the contrary, as noted above, three of the ODX low‐risk patients (who had received endocrine therapy alone) reclassified as high‐risk by CAB showed distant recurrence within 5 years.

As has been noted, the relatively small sample size is a shortcoming of the current study, particularly because this prevented rigorous statistical analyses of the data. In addition, since all ODX high‐risk patients were given chemotherapy, the outcomes of ODX high‐risk patients could not be analyzed or extrapolated in the absence of data on such patients who had not received chemotherapy. Moreover, since very few Indian patients are in the cohort, ethnicity and nationality‐based extrapolations could not be drawn. With the caveat of these limitations, our results indicate that the performance of CAB is as good as ODX with respect to the actual patient outcomes. Efforts are underway to expand this study further.

## 5 CONCLUSION

The analysis presented above reiterates that the risk stratification of CAB into low‐ and high‐risk groups was of good accuracy as measured by NPV when compared with the patient outcomes in 5 years. These results also demonstrate excellent concordance with ODX low‐risk categories with better overall concordance than has been reported with most other prognostic tests. Having been validated in Indian and Caucasian patients, CAB is an excellent and affordable alternative to ODX, particularly in India and other Asian countries.

## CONFLICT OF INTERESTS

All authors except AB are employees of OncoStem Diagnostics Pvt. Ltd., which developed CanAssist Breast. MMB is a coinventor on a patent application related to this article. All other authors have no competing interest to declare.

## AUTHOR CONTRIBUTIONS

MMB conceived and designed the study. AKS and AG analyzed, interpreted the data, and drafted the manuscript. SM had performed the preliminary analysis of a subset of the current cohort. CPSV was involved in data acquisition, performed the experiments, and helped with analysis. CB performed all histopathological analyses. AB helped with sample acquisition. All authors have read and approved the final manuscript.

## Supporting information

Table S1‐S2

## Data Availability

The data sets used and/or analyzed during the current study are available from the corresponding author on reasonable request.
