# Supplementary material for: Comparison of breast cancer prognostic tests CanAssist Breast and Oncotype DX
Source: Cancer Med. 2020 Oct 7;9(21):7810–8. doi: 10.1002/cam4.3495 (PMC7643688; doi:10.1002/cam4.3495)
Supplement: Supplementary file 1 — Table S1‐S2 [file CAM4-9-7810-s001.docx]

**Supplementary Table S1:** **Stratification of the by ODX, ODX with TAILORx cut offs (ODX-Tx) and CAB of the node negative (N0) and node positive (N+) sub-cohorts.** Patients who received endocrine therapy alone are designated as ET and those that received both endocrine therapy and chemotherapy are designated as ET + CT.

|  | | | N0 | | | N+ | | |
| --- | --- | --- | --- | --- | --- | --- | --- | --- |
|  |  |  | Total | ET | ET + CT | Total | ET | ET + CT |
| Total Cohort | | Total | 93 | 68 | 25 | 16 | 8 | 8 |
|  |  | Recurred | 7 | 5 | 2 | 2 | 2 | 0 |
| ODX | Low Risk | Total | 61 | 56 | 5 | 7 | 6 | 1 |
|  |  | Recurred | 5 | 5 | 0 | 2 | 2 | 0 |
|  | Intermediate Risk | Total | 27 | 12 | 15 | 7 | 2 | 5 |
|  |  | Recurred | 2 | 0 | 2 | 0 | 0 | 0 |
|  | High Risk | Total | 5 | 0 | 5 | 2 | 0 | 2 |
|  |  | Recurred | 0 | 0 | 0 | 0 | 0 | 0 |
| ODX-Tx | Low Risk | Total | 84 | 68 | 16 | 14 | 8 | 6 |
|  |  | Recurred | 6 | 5 | 1 | 2 | 2 | 0 |
|  | High Risk | Total | 9 | 0 | 9 | 2 | 0 | 2 |
|  |  | Recurred | 1 | 0 | 1 | 0 | 0 | 0 |
| CAB | Low Risk | Total | 81 | 57 | 24 | 10 | 5 | 5 |
|  |  | Recurred | 5 | 3 | 2 | 1 | 1 | 0 |
|  | High Risk | Total | 12 | 11 | 1 | 6 | 3 | 3 |
|  |  | Recurred | 2 | 2 | 0 | 1 | 1 | 0 |

**Supplementary Table S2:** CAB stratification and outcomes of the patients who did not receive chemotherapy and were classified into low risk by ODX and ODX with TAILORx cut offs (ODX-Tx) respectively. All patients classified as high risk by ODX received chemotherapy.

| Chemo-naïve subcohort | ODX Low Risk | | | | ODX-Tx Low Risk | | | |
| --- | --- | --- | --- | --- | --- | --- | --- | --- |
|  | All samples (n=62) | | N0 (n=56) | | All samples (n=76) | | N0 (n=68) | |
|  | Number | Recurred | Number | Recurred | Number | Recurred | Number | Recurred |
| CAB Low Risk | 51 (82.3%) | 4 (7.8%) | 48 (85.7%) | 3 (6.25%) | 62 (81.6%) | 4 (6.5%) | 57 (83.8%) | 3 (5.3%) |
| CAB High Risk | 11 (17.7%) | 3 (27.3%) | 8 (14.3%) | 2 (25%) | 14 (18.4%) | 3 (21.4%) | 11 (16.2%) | 2 (18.2%) |
| Total | 62 | 7 (11.3%) | 56 | 5 (8.9%) | 76 | 7 (9.21%) | 68 | 5 (7.4%) |
